# *Bursatella leachii* Purple Ink Secretion Concentrate Exerts Cytotoxic Properties against Human Hepatocarcinoma Cell Line (HepG2): In Vitro and In Silico Studies

**DOI:** 10.3390/molecules27030826

**Published:** 2022-01-26

**Authors:** Zeyad I. Alehaideb, Anuradha Venkatraman, Mahadev Kokane, Syed Ali Mohamed, Saranya Rameshbabu, Rasha S. Suliman, Sahar S. Alghamdi, Hamad Al-Eidi, Bandar Alghanem, Maha-Hamadien Abdulla, Sabine Matou-Nasri

**Affiliations:** 1 King Abdullah International Medical Research Center, King Saud bin Abdulaziz University for Health Sciences, Riyadh 11481, Saudi Arabia; alehaidebze1@NGHA.MED.SA (Z.I.A.); sulimanr@ksau-hs.edu.sa (R.S.S.); ghamdisa@ksau-hs.edu.sa (S.S.A.); aleidiha@NGHA.MED.SA (H.A.-E.); GhanemBa@NGHA.MED.SA (B.A.); 2 PG & Research Department of Biochemistry, Mohamed Sathak Arts and Science College, Tamil Nadu 600119, India; vanuradha.2003@gmail.com (A.V.); mahadevciba@gmail.com (M.K.); 3 Central Institutes of Fisheries Nautical and Engineering Training, Tamil Nadu 600013, India; 4 PG & Research Department of Biotechnology, Mohamed Sathak Arts and Science College, Tamil Nadu 600119, India; syedmicro555@gmail.com (S.A.M.); saranyasundar2012@gmail.com (S.R.); 5 College of Pharmacy, King Saud bin Abdulaziz University for Health Sciences, Riyadh 11481, Saudi Arabia; 6 Colorectal Research Chair, Department of Surgery, College of Medicine, King Khalid University Hospital, King Saud University, Riyadh 11472, Saudi Arabia; mabdulla@ksu.edu.sa

**Keywords:** ADME, apoptosis, *Bursatella leachii* ink, caspase, target prediction, TP53, liver cancer

## Abstract

Liver cancer is a leading cause of cancer death globally. Marine mollusc-derived drugs have gained attention as potential natural-based anti-cancer agents to overcome the side effects caused by conventional chemotherapeutic drugs during cancer therapy. Using liquid chromatography-mass spectrometry, the main biomolecules in the purple ink secretion released by the sea hare, named *Bursatella leachii* (*B. leachii*), were identified as hectochlorin, malyngamide X, malyngolide S, bursatellin and lyngbyatoxin A. The cytotoxic effects of *B. leachii* ink concentrate against human hepatocarcinoma (HepG2) cells were determined to be dose- and time-dependent, and further exploration of the underlying mechanisms causing the programmed cell death (apoptosis) were performed. The expression of cleaved-caspase-8 and cleaved-caspase-3, key cysteine-aspartic proteases involved in the initiation and completion of the apoptosis process, appeared after HepG2 cell exposure to the *B. leachii* ink concentrate. The gene expression levels of pro-apoptotic *BAX*, *TP53* and *Cyclin D1* were increased after treatment with the *B. leachii* ink concentrate. Applying in silico approaches, the high scores predicted that bioactivities for the five compounds were protease and kinase inhibitors. The ADME and cytochrome profiles for the compounds were also predicted. Altogether, the *B. leachii* ink concentrate has high pro-apoptotic potentials, suggesting it as a promising safe natural product-based drug for the treatment of liver cancer.

## 1. Introduction

Liver cancer is the sixth most frequently diagnosed cancer and third leading cause of cancer death globally, with a high incidence observed in Asian and African countries [1]. The variable geographical distribution of liver cancer overlaps with the geographic incidence of viral hepatitis (i.e., hepatitis B and C viruses) and of the human immunodeficiency virus (HIV) [2]. This viral infection results in the onset of liver cancer and the progression from chronic hepatitis, liver cirrhosis to heterogeneous hepatocellular carcinoma (HCC) [3]. The onset of liver cancer can also be due to aging, exposure to toxic compounds, autoimmunity and metabolic diseases [4]. Conventional treatments such as surgery, radiotherapy and chemotherapy, as well as gene- and immune-based therapeutic drugs are currently used [5]. Cytotoxic chemotherapy is not the first-line treatment (i.e., protein kinase inhibitor, Sorafenib) for HCC, the main type of primary liver cancer, which is a chemotherapy-refractory tumour [6]. However, there is ongoing discovery of natural bioactive compounds as neo-adjuvant agents, which inhibit liver cancer cell growth and enhance liver cancer prevention, as well as overcome hepatotoxicity side effects from conventional therapy and liver cancer recurrence [7].

An alternative treatment for liver cancer prior to transplantation is urgently required. Natural product extracts have been investigated for their bioactive compounds with anti-proliferative activity and pro-apoptotic effects, revealed by tyrosine kinase inhibition [8] and by caspase activation, cell cycle- and apoptosis-related gene up-regulation [9]. The main advantage of the induction of apoptosis is the absence of the inflammatory reaction triggered by necrotic cells [10]. Some natural bioactive compounds with anti-inflammatory and anti-angiogenic effects, which prevent cancer progression, have been discovered [11].

Molluscs are the second largest animal phylum on earth and provide a rich source of medicinal natural bioactive molecules [12]. The opisthobranch molluscs are a subclass of the *Gastropoda* family *Aplysiidae*, order *Anaspide*, genus and species *Bursatella (B.) leachii*, commonly known as sea hares [13]. Sea hare-derived bioactive compounds with anti-cancer activity, including soblidotin (dolastatin 10 derivative), synthadotin/ILX_651_, cemadotin and kahalalide F, have been in clinical trials, and brentuximab vedotin Adcetris^®^ (dolastatin 10), an antibody drug conjugate, has been approved by the Food and Drug Administration for the treatment of Hodgkin lymphoma and systemic anaplastic large cell lymphoma [14,15,16]. Similar to a squid, sea hares release a purple ink to fend off predators which contains secondary metabolites with potential cytotoxicity [16]. We previously reported *B. leachii* purple ink-derived anti-HIV protein [17], 7,9-di-tert-butyl-1-oxaspiro [4,5], deca-6,9-diene-2,8-dione and digoxigenin acetate as potent anti-inflammatory compounds [18]. However, *B. leachii* purple ink-derived concentrate, including the anti-inflammatory compounds identified, has not been studied for its potential anti-cancer activity. We chemically analysed *B. leachii* ink concentrate to identify secondary metabolites and evaluated the potential cytotoxic effects of a crude ink concentrate of *B. leachii* against the growth of the human hepatocellular carcinoma (HCC) cell line HepG2. Protein and gene expression levels of apoptosis and cell cycle regulatory markers in the *B. leachii* ink concentrate-treated HepG2 cells were assessed and several biological target predictions were performed.

## 2. Results

### 2.1. Chemical Identification of B. leachii Ink Concentrate Using Liquid Chromatography-Quadrupole Time of Flight (LC-QTOF)

**Figure 1:**
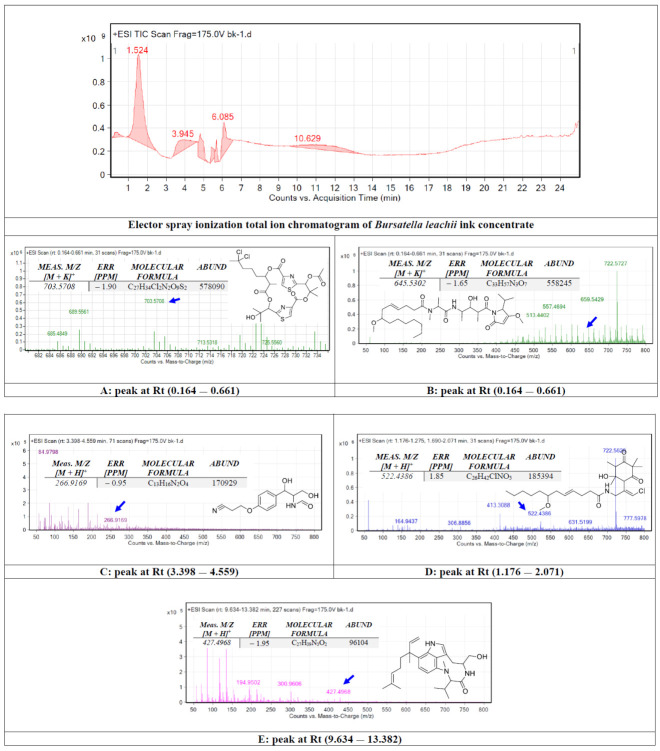
Base peak chromatogram of *Bursatella leachii* ink concentrate. Tentatively identified secondary metabolites are hectochlorin (**A**) [19], malyngamide X (**B**) [20], bursatellin (**C**) [21], malyngamide S (**D**) [22] and lyngbyatoxin A (**E**) [23]. Means *m*/*z* implies measured *m*/*z*.

The crude ink concentrate of the *B. leachii* was subjected to total ion current spectra raw data (See Figure 1). Qualitative and quantitative analysis software from the data analysis program MassHunter (Agilent Technologies) were also used. After conducting a mass screening on the spectra (Figure 1), the chemical features were extracted from the LC-QTOF data using the Molecular Features Extraction algorithm and the recursive analysis workflow. Features were extracted by screening the detected nodes at various retention times per minute, with a minimum intensity of 6000 counts, and aligned with previously detected compounds considering adducts ([M + K]^+^ and [M − H]^−^). The tentatively identified compounds were hectochlorin (A), malyngamide X (B), bursatellin (C), malyngamide S (D) and lyngbyatoxin A (E).

### 2.2. Cytotoxic Effect of the B. leachii Ink Concentrate

**Figure 2:**
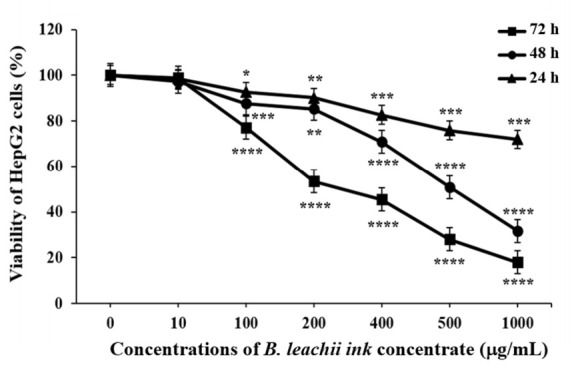
Effects of B. leachii concentrate on HepG2 cell viability. The cell viability was determined using CellTiter-Glo^®^ kit and expressed as percentage of the control, the untreated cell viability, corresponding to 100%. * *p* < 0.05, ** *p* < 0.01, *** *p* < 0.001, and **** *p* < 0.0001 signify a statistically significant difference compared with the control, from three independent experiments.

The effect of *B. leachii* ink concentrate on HepG2 cell proliferation was tested at different concentrations and time periods. The increasing concentrations (from 10 to 1000.0 μg/mL) of the *B. leachii* ink concentrate and the lengthening of the exposure times (from 24 to 72 h) resulted in a decrease in the viability of the HepG2 cells, based on the ATP generated by the living cells, compared to the control cells, which described a dose- and time-dependent effect (Figure 2). The half-maximal inhibitory concentration (IC_50_) values of *B. leachii* ink concentrate required in order to inhibit 50% of HepG2 cell growth were determined at each exposure time. The treatment of HepG2 cells with *B. leachii* ink concentrate for 72 h of exposure displayed the lowest IC_50_ value of 242.9 µg/mL, followed by 48 h exposure with an IC_50_ value of 447.5 µg/mL, and the IC_50_ value for 24 h was more than 1000 µg/mL of *B. leachii* ink concentrate.

### 2.3. Induction of Apoptosis by B. leachii Ink Concentrate

**Figure 3:**
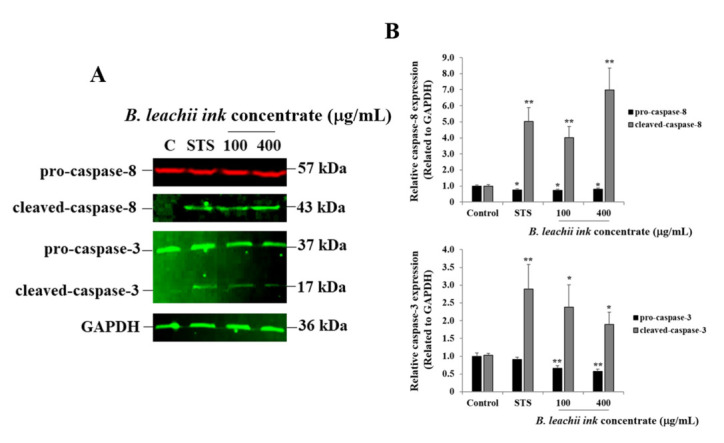
*B. leachii* ink concentrate triggers caspase-8 and caspase-3 cleavage in HepG2 cells. (**A**) Representative Western blot gels showing the detection of cleaved-caspase-8 and of cleaved-caspase-3 in HepG2 cells after 24 h of treatment with 100 and 400 g/mL of *B. leachii* ink concentrate along with 1 M STS, used as a positive control. Quasi-absence of the cleavage of caspase was observed in untreated HepG2 cells, the control (C). (**B**) Bar graphs indicating the relative expression levels of caspase-8 (top) and of caspase-3 (bottom), calculated as a ratio of the expression to GAPDH, used as a loading control. * *p* < 0.05 and ** *p* < 0.01 signify a statistically significant difference compared with the control, from three independent experiments.

As one of the cell death mechanisms, the potential induction of apoptosis in HepG2 cells treated with 100 µg/mL and 400 µg/mL of the *B. leachii* ink concentrate was investigated using Western blot analysis. After 24 h of exposure, the expression levels of pro-apoptotic proteins, such as the cleaved caspase-8 (key enzyme prompting extrinsic apoptotic pathway) and cleaved-caspase-3 (key enzyme resulting in apoptosis), were evaluated in the *B. leachii* ink concentrate-treated cells compared with the untreated cells. Used as a positive control, staurosporine (STS) led to a cleavage of both caspase-8 and caspase-3 in HepG2 cells, and, as expected, a quasi-absence of cleaved-caspase-8 and cleaved-caspase-3 expression was detected in the untreated cells (Figure 3). A cleavage of both caspase-8 and caspase-3 was observed in *B. leachii* ink concentrate-treated HepG2 cells (Figure 3). Higher levels of expression of the cleaved-caspase-8 were observed in the HepG2 cells treated with 400 µg/mL of *B. leachii* ink concentrate compared with the cells treated with 100 µg/mL of *B. leachii* ink concentrate (Figure 3). A similar expression level of cleaved-caspase-3 was noticed in the HepG2 cells exposed to the two tested concentrations of *B. leachii* ink concentrate (Figure 3).

### 2.4. Modulation of Gene Expression Levels of Apoptotic and Cell Cycle Regulatory Genes by B. leachii Ink Concentrate

**Figure 4:**
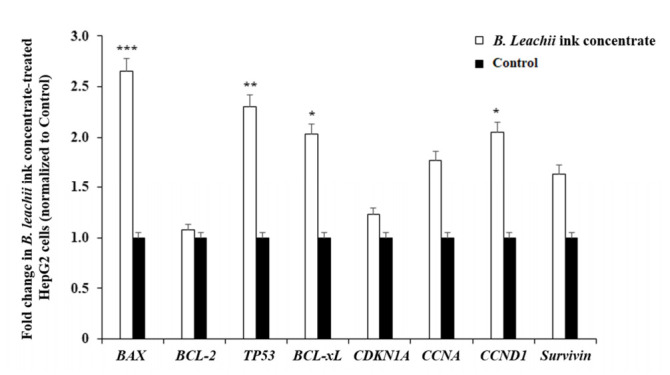
Effects of *B. leachii* ink concentrate on apoptosis and cell cycle regulatory gene expression detected in HepG2 cells. The bar graph shows the relative gene expression of apoptotic (Bax, Bcl2, Bcl-xL, Survivin), tumour suppressor TP53 and cell cycle (cyclin A, cyclin D1, cyclin-dependent kinase inhibitor CDKN1A) regulatory transcripts determined by RT-qPCR analysis in HepG2 cells treated with 400 g/mL of *B. leachii* ink concentrate, as compared with the basal level of gene expression monitored in untreated HepG2 cells, the control. * *p* < 0.05, ** *p* < 0.01, and *** *p* < 0.001 signify a statistically significant difference compared with the control, from three independent experiments.

The pro-apoptotic effect of *B. leachii* ink concentrate on the HepG2 cells was evaluated by monitoring the expression levels of apoptosis and cell cycle-related genes, including *BCL-2*, *BCL-xL*, *TP53*, *BAX*, *CDKN1A*, *CCNA* (Cyclin A), *CCND1* (Cyclin D1) and *Survivin* using reverse transcription-quantitative polymerase chain reaction (RT-qPCR). The treatment of the HepG2 cells with 400 µg/mL of *B. leachii* ink concentrate significantly enhanced the expression levels of the pro-apoptotic genes *BAX* (2.6-fold, *p* = 0.000012), *TP53* (2.3-fold, *p* = 0.00023) and *CCND1* (2.1-fold, *p* = 0.0012), compared with the basal gene expression level detected in the untreated cells (Figure 4). A significant up-regulation of the gene expression level of the anti-apoptotic *BCL-xL* (2.0-fold, *p* = 0.0043) was noticed in the *B. leachii* ink concentrate-treated HepG2 cells (Figure 4). Of note, the up-regulation of the pro-apoptotic *CCNA* (1.77-fold) and anti-apoptotic *Survivin* (1.64-fold) gene expression levels remained non-significant (Figure 4).

### 2.5. B. leachii Ink Concentrate Bioactivity Predictions

In this study, we sought to determine the bioactivity score of each bioactive metabolite identified in the *B. leachii* ink concentrate. These bioactivity predictions provided more information about which molecule in the ink concentrate could contribute to the observed anti-cancer activity. The bioactivity score of the five bioactive molecules was investigated using the PASS online webserver. Our results showed that hectochlorin and malyngamide S had the highest bioactivity scores (Table 1), with Pa 0.933 and Pa 0.747, respectively, suggesting a promising anti-neoplastic activity for these two molecules. Additionally, the anti-cancer activity of *B. leachii* ink concentrate could be attributed to the presence of hectochlorin and malyngamide S metabolites. The remaining molecules exhibited lower bioactivity scores, and no predicted score was identified for bursatellin.

### 2.6. Molecular Target Predictions of B. leachii Ink Concentrate

Molinspiration was used to investigate the possible molecular targets that could mediate the observed and predicted anti-cancer activity. Each bioactive molecule was evaluated as a G protein-coupled receptor (GPCR) ligand, ion channel modulator, kinase inhibitor, nuclear receptor ligand and protease and enzyme inhibitor. Interestingly, almost all of the five molecules exhibited a positive bioactivity score as protease inhibitors, and malyngamide X, malyngamide S and lyngbyatoxin A had the highest scores (0.46, 0.32 and 0.36, respectively). Malyngamide X, malyngamide S and lyngbyatoxin A had high bioactivity scores as enzyme inhibitors with values of 0.32, 0.32 and 0.35, respectively. Other possible targets such as the ion channels, GPCR and kinase inhibition were seen with malyngamide X, malyngamide S and lyngbyatoxin A, suggesting that these bioactive molecules could regulate several molecular targets. Additional target mapping was conducted with SwissTargetPrediction and the results were comparable with the Molinspiration webserver with a high probability of targeting proteases and kinases (Table 2).

### 2.7. Pharmacokinetics Absorption, Distribution, Metabolism and Excretion (ADME) Predictions and Cytochrome (CYP) P450 Enzyme Inhibition Profiling

To assess the potential pharmaceutical properties of the *B. leachii* ink concentrate-derived bioactive molecules, SwissADME (Swiss Institute of Bioinformatics, Lausanne, Switzerland) was used to evaluate several parameters important for drug discovery. Of the five bioactive metabolites, malyngamide S, bursatellin and lyngbyatoxin A demonstrated a molecular weight of fewer than 500 daltons. All the compounds that demonstrated a high lipophilicity, except for bursatellin, exhibited a low Log *p* value. The solubility of the compounds was poor, with the exception of bursatellin, which demonstrated excellent solubility (Log S −2.39). Most of the compounds were predicted to have a peripheral effect (no blood–brain barrier (BBB) penetration except for lyngbyatoxin A) and high gastrointestinal (GI) absorption (Table 3).

We determined and qualitatively predicted the possibility of CYP enzyme inhibition that could be associated with these bioactive molecules using the SWISS webserver. Our results showed that hectochlorin and bursatellin did not exhibit any CYP enzyme inhibition. Malyngamide X demonstrated only two inhibitions for CYP2C19 and CYP3A4, and the malyngamide S was predicted to inhibit CYP2C19, CYP2D6 and CYP3A4 enzymes. Lyngbyatoxin A inhibited three CYP enzymes, including CYP2C19, CYP2C9 and CYP3A4 (Table 4).

## 3. Discussion

Intensive exploration of the marine ecosystem has provided a valuable source of diverse bioactive compounds. Recently, the purple ink concentrate released by the sea hare *B. leachii* was studied, and anti-HIV and anti-inflammatory activities were identified [17,18], two prominent properties required for a potential liver cancer treatment. To widen the biological activities of this *B. leachii* purple ink-derived concentrate as a promising natural neo-adjuvant for the treatment of liver cancer, we investigated its potential cytotoxic effects against HCC HepG2 cells and established molecular target and pharmacokinetic predictions of the identified metabolites-derived *B. leachii* purple ink concentrate.

In this study, the chemical analysis was performed using high-resolution Q-TOF analysis, which supports the tentative identification of the chemicals more accurately and the comparison with previous structure identification studies for the *B. leachii* ink concentrate biomolecules. For example, the *m*/*z* value at retention time (0.164–0.661) was correlated with the parent compound hectochlorin [19], with *m*/*z* [M + K]^+^ 703.5708 daltons and a molecular formula of [C_27_H_34_Cl_2_N_2_O_9_S_2_]^+^, in the positive ion mode [M + H]^+^ *m*/*z* 666.236 and [M − H]^–^ with *m*/*z* 664.163 daltons in the negative mode, indicating that the compound had a molecular weight of 665.603 g·mol^−1^. The *m*/*z* value at retention time (0.164–0.661) was correlated with the parent compound malyngamide X [20], with *m*/*z* [M + K]^+^ 646.5302 daltons and a molecular formula of [C_33_H_57_N_3_O_7_]^+^, in the positive ion mode [M + H]^+^ *m*/*z* 607.420 and [M − H]^–^ with *m*/*z* 606.822 daltons in the negative mode, indicating that the compound had a molecular weight of 607.420 g·mol^−1^. The *m*/*z* value at retention time (3.398–4.559) was correlated with the parent compound bursatellin [21], with *m*/*z* [M + 2H]^+^ 266.9169 daltons and a molecular formula of [C_13_H_16_N_2_O_4_]^+^, in the positive ion mode [M + H]^+^ *m*/*z* 264.111 and [M − H]^–^ with *m*/*z* 263.210 daltons in the negative mode, indicating that the compound had a molecular weight of 264.277 g·mol^−1^. The *m*/*z* value at retention time (1.176–2.071) was correlated with the parent compound malyngamide S [22], with *m*/*z* [M + K]^+^ 522.4386 daltons and a molecular formula of [C_26_H_42_ClNO_5_]^+^, in the positive ion mode [M + H]^+^ *m*/*z* 483.279 and [M − H]^–^ with *m*/*z* 482.109 daltons in the negative mode, indicating that the compound had a molecular weight of 484.069 g·mol^−1^. The *m*/*z* value at retention time (9.634–13.382) was correlated with the parent compound lyngbyatoxin A [23], with *m*/*z* [M − CH_3_]^+^ 426.4968 daltons and a molecular formula of [C_27_H_39_N_3_O_2_]^+^, in the positive ion mode 437.304 and [M − H]^−^ with *m*/*z* 437.304 daltons in the negative mode, indicating that the compound had a molecular weight of 437.617 g·mol^−1^.

The human HCC cell line HepG2 exposed to the *B. leachii* purple ink secretion concentrate led to an inhibition of the cell proliferation in a dose- and time-dependent manner. An induction of apoptosis in *B. leachii* ink concentrate-treated HepG2 cells was observed at intermediate concentrations (100 and 400 μg/mL) of *B. leachii* ink concentrate after 24 h exposure. *B. leachii* ink concentrate added to the HepG2 cells for 72 h of treatment resulted in the lowest IC_50_ value of 242.9 µg/mL compared with IC_50_ values determined after 24 and 48 h of exposure. A study conducted by Suntornchashwej and colleagues [19] reported the cytotoxicity exhibited by the ethyl acetate-derived ink extract of *B. leachii* against the human small cell lung cancer (NCI-H187), oral human epidermoid carcinoma (KB) and breast cancer (BC) cell lines with half-maximal effective dose (ED_50_) values of 16.2, 7.2 and 6.6 µg/mL, respectively. The protective effect of *B. leachii* ink extract against neuroblastoma cell line SH-SY5Y pre-treated with hydrogen peroxide and against microglia cells stimulated by a bacterial lipopolysaccharide was studied [24]. *B. leachii* ink extract was effective against microglia cells by decreasing the intracellular nitric oxide production with an IC_50_ value of 5.74 μg/mL; however, the SH-SY5Y cells had no cell response to the concentration of the *B. leachii* ink extract studied. The determination of different IC_50_ values reflecting the anti-proliferative activity of *B. leachii* ink extract indicate the specificity of the ink extract to exert cytotoxicity against various cancer cell lines.

Similarly, the anti-cancer potential of *B. leachii* ink concentrate at 100 and 400 µg/mL through apoptosis induction in HepG2 cells treated for 24 h was confirmed using Western blot technology by quantitatively detecting the expression of the most important pro-apoptotic proteins, namely the initiator caspase-8 and the effector/executioner caspase 3. Apoptosis is initiated and achieved by the cleavage of the initiator and executioner pro-caspases into cleaved-caspases, the activated form of the enzymes. In the present study, the cleavage of caspase-8 and caspase-3 was observed in the HepG2 cells after 24 h of cell exposure to *B. leachii* ink concentrate. An equal level of cleaved-caspase-3 was observed at both concentrations (100 and 400 µg/mL) of *B. leachii* ink concentrate, confirming the slight decrease in HepG2 cell growth noticed at similar conditions. Tested at the same intermediate concentrations, a concomitant cleavage of pro-caspase-3 would be expected after 72 h of exposure, due to the decrease in the *B. leachii* ink concentrate-treated HepG2 cell growth. However, the degree of pro-caspase-8 cleavage increased with the concentrations of *B. leachii* ink concentrate, confirming the role of caspase-8 as the initiator of apoptosis. In addition, the expression of cleaved-caspase-8 and cleaved-caspase-3 detected in *B. leachii* ink concentrate-treated HepG2 cells may reveal that the induction of apoptosis occurs via the activation of the extrinsic death receptor pathway by transmitting the death signal from the cell surface to the intracellular signalling pathways through the tumour necrosis factor receptor gene family [25]. Active caspase-8 either initiates apoptosis directly by cleaving pro-caspase-3 into activated cleaved-caspase-3 or through the mitochondria by the cleavage of BID to induce cell death [26]. An investigation of the involvement of the mitochondrial-dependent intrinsic apoptosis pathway, such as monitoring the mitochondrial membrane potential or release of cytochrome and other apoptotic proteins, in *B. leachii* ink concentrate-treated HepG2 cells would be of interest.

The quantitative expression of cell cycle and apoptotic regulatory genes in the HepG2 cells treated with 400 µg/mL of *B. leachii* after 48 h of exposure was analysed using RT-qPCR. The expressions of the predominant pro-apoptotic *BAX*, *TP53* and *CCND1* (Cyclin D1) genes were significantly up-regulated over the increased expression of the anti-apoptotic *BCL-xL* gene. The gene expression of the anti-apoptotic *BCL-2*, belonging to the main Bcl-2 family protein regulators of apoptosis that are endowed with pro- and anti-apoptotic activities, was not modulated even after the HepG2 cell exposure to *B. leachii* ink concentrate. The tumour suppressor p53 is a transcriptional protein activated by a variety of oncogenic/hyperproliferative stimuli, including DNA damage or chemotherapeutic drugs, and can regulate downstream pro-apoptotic (i.e., *BAX* and p53 upregulated modulator of apoptosis *PUMA* up-regulation) and anti-apoptotic genes (i.e., *BCL-2* repression) [27,28]. Of note, concerning the observed up-regulation of *BCL-xL* and *CCND1* gene expression, both the *BCL-xL* and *CCND1* promoters contain signal transducer and activators of transcription (STAT) binding sites [29], suggesting a possible STAT transcription factor activation in *B. leachii* ink concentrate-treated HepG2 cells. The protein p53 located in the cytosol induces the activation of pro-apoptotic Bax by protein–protein interactions and with Bcl-xL and Bcl-2 by p300/CBP binding [30]. Bax protein homodimerization results in pore formation in the outer mitochondrial membrane, facilitating the release of pro-apoptotic proteins, and Bax-Bcl-2 heterodimerization results in the neutralization of Bcl-2 anti-apoptotic activity [25]. The elevated gene expression levels of *TP53* and *BAX* in *B. leachii* ink concentrate-treated HepG2 cells endorses that cell death might be due to p53-dependent apoptosis.

The analysis of the bioactivity predictions indicates that of the five identified compounds, hectochlorin and malyngamide S had the highest scores as anti-neoplastic agents. This is important for lead optimization and development. The prediction supports the observed anti-cancer activity of the *B. leachii* ink concentrate that could be attributed to the presence of hectochlorin and malyngamide S. The molecular target predictions established from the *B. leachii* ink concentrate suggest the involvement of proteases and kinase inhibitors as potential targets that could explain the observed up-regulation of several apoptotic markers. Several studies reported the modulation of proteases and kinases by marine bioactive molecules, which induced apoptosis in cancer cells [23,31,32,33].

The in silico ADME predictions for the identified molecules are useful for the potential use of these compounds as a lead for the discovery of a novel anti-cancer therapy. The ADME properties data showed that most of the bioactive molecules have acceptable pharmaceutical properties and follow Lipinski’s rule-of-five for drugability related to absorption/permeation, molecular weight and solubility [34,35], except for hectochlorin, which had two violations of this rule. Moreover, the CYP enzyme inhibition profile suggests that some of the compounds could inhibit CYP2C19 and CYP3A4 activities. However, this inhibition profile could be overcome with future optimization of the lead compound.

## 4. Materials and Methods

### 4.1. Collection of B. leachii Ink Concentrate

The adult *B. leachii* sea hares were collected from intertidal waters of Pulicat lake, position Lat. 13.452523° N Long. 80.319133° E +/− 0.03° N/E, and brought to the laboratory in live condition. The accession number was M-1697, obtained from the Zoological Survey of India, Marine Biology Regional Centre (MBRC), Chennai, India and dated 23 July 2015. Obtained by disturbing the *B. leachii*, the purple fluid ink was filtered through Whatman^®^ filter paper (Sigma-Aldrich, St. Louis, MO, USA). All the aqueous ink-derived samples were centrifuged at 15,000× *g* for 15 min as described by Vennila and colleagues [36] and the supernatant was kept and lyophilized to purple ink residue using a freeze dryer and stored at 4 °C for further use.

### 4.2. Chemicals and Reagents

Dulbecco’s Modified Eagles Medium (DMEM), foetal bovine serum (FBS), penicillin, streptomycin and l-glutamine were obtained from Gibco™ (Waltham, MA, USA). Staurosporine (STS) (>99%) was obtained from Santa Cruz biotechnology (Dallas, TX, USA). High-purity methanol (99.9%) was procured from Honeywell (Charlotte, NC, USA). Formic acid (>95.0%) was purchased from Sigma-Aldrich. Ultrapure water was produced using a Millipore (Billerica, MA, USA) system with a resistivity reading of 18.2 MΩ·cm at 25 °C.

### 4.3. Chemical Analysis Using LC-QTOF

The fingerprinting of *B. leachii* secretion of aqueous ink concentrate was performed using the Agilent (Santa Clara, CA, USA) 1260 Infinity high performance liquid chromatography system coupled to Agilent 6530 Q-TOF. The analysis was performed using an Agilent SB-C18 column (4.6 mm × 150 mm, 1.8 μm) with the following elution gradient: 0–2 min, 5% B; 2–17 min, 5–100% B; 17–21 min, 95% B; 21–25 min, 5% B, using mobile phase A (0.1% formic acid in water) and mobile phase B (0.1% formic acid in methanol). The injection volume was 10 µL and the flow rate was set at 250 µL/min. The scanning range was set at 50–800 (*m*/*z*) and the remaining parameters were set as follows: gas temperature at 300 °C, gas flow at 8 L/min, nebulizer pressure at 35 psi, sheath gas temperature at 350 °C and sheath gas flow rate at 11 L/min. The data were generated by the Agilent MassHunter qualitative analysis software (version B.06.00).

### 4.4. Cell Line and Culture Medium

The human HCC cell line HepG2 (#HB-8065, American Type Culture Collections, Manassas, VA, USA) was cultured in DMEM, supplemented with 10% FBS, 100 IU/mL of penicillin, 100 μg/mL of streptomycin and 2 mM of l-glutamine. The cells were maintained at 37 °C in a 5% humidified CO_2_ incubator.

### 4.5. Cell Proliferation Assay

The HepG2 cells (5000/0.1 mL) were seeded in white flat-bottom 96-well plates (Costar^®^, Thermo Fisher Scientific, Waltham, MA, USA). After 24 h of incubation, the cells were independently treated 3 times in triplicate with various concentrations of *B. leachii* ink concentrate (10, 100, 200, 400, 500 and 1000 μg/mL). The wells containing the culture media and the cells without treatment served as blank and control, respectively. The cell proliferation was measured after 24, 48 and 72 h using the CellTiter-Glo^®^ Luminescent Cell Viability Assay (Promega Corporation Inc., Fitchburg, WI, USA) and we determined the half-maximal inhibitory concentration (IC_50_) value as described in [37].

### 4.6. Western Blot Analysis

The HepG2 cells (5 × 10^5^/mL) were seeded in Nunc™ 12-well plates (Thermo Fisher Scientific, Inc.). After 24 h of incubation, the cells were treated in triplicate with *B. leachii* ink concentrate at 100 and 400 µg/mL along with 1 µM STS, used as a positive control. Western blot technology and image analysis were employed as described in [38]. Polyvinylidene difluoride membranes (Millipore, Thermo Fisher Scientific) were probed with (1:1000 dilution) mouse anti-pro/cleaved-caspase-3, rabbit anti-pro-caspase-8 and mouse anti-cleaved-caspase-8 antibodies (Cell Signalling Technology, Danvers, MA, USA) and mouse anti-GAPDH antibody (Abcam, Cambridge, UK).

### 4.7. Gene Expression Analysis

The HepG2 cells (1.5 × 10^6^) were seeded in Nunc™ 6-well plates. After 24 h of incubation, the cells were treated in triplicate with or without 400 µg/mL of *B. leachii* ink concentrate and incubated for 48 h. From the total RNA ink concentrate ion to reverse-transcribed cDNA, RT-qPCR was performed as described in [39]. The relative quantifications of the mRNA expression level for the target genes are listed in the Table 1.

### 4.8. Activity Prediction Using PASS Online Webserver

The anti-neoplastic activity of the bioactive metabolites identified from *B. leachii* ink concentrate was assessed using Pass online webserver (Way2Drug, Moscow, Russia Version 2.0) [40]. For each compound, the SMILES (Simplified Molecular Input Line Entry System) was generated and entered in the webserver to perform the assessment. The results were classified based on the compound probability of being active (Pa) and inactive (Pi) for the specified activity.

### 4.9. Target Predictions Using Molinspiration and SwissTargetPrediction Tools

To investigate the possible molecular targets for these metabolites identified from *B. leachii* ink concentrate, Molinspiration (Molinspiration Cheminformatics, Slovenský Grob, Slovakia) [41] and SwissTargetPrediction (Swiss Institute of Bioinformatics, Lausanne, Switzerland) were used [42]. For both webservers, SMILES was applied to generate the data. The Molinspiration web server produced a score that reflected the bioactivity of the compound. Positive values indicated the highest probability that the compound was active at the molecular target. For the SWISS target predictions, a general mapping of the possible molecular targets was provided for any compound, which facilitated the identification of the biological targets of uncharacterized molecules [39,40,41,42].

### 4.10. Pharmacokinetic ADME Predictions and Cytochrome P450 Profiling Using SWISS Tool

The pharmacokinetics concerning the ADME of the identified bioactive metabolites from *B. leachii* ink concentrate was explored using the SwissADME web server (Swiss Institute of Bioinformatics, Lausanne, Switzerland), which provided detailed, fast, in silico predictions of the pharmaceutical profiles of the bioactive compounds [43]. The selected ADME parameters for the analysis were molecular weight, lipid solubility (Log P), water solubility (Log S), BBB penetration and GI absorption. After data generation, the results were compared with the established drug-likeness properties (rule-of-five, ROF) important for drug discovery [44].

Additional investigations were conducted to assess the CYP inhibition profile of the bioactive molecules using the SWISS web server. Each compound was evaluated against several CYP enzymes, including CYP1A2, CYP2C19, CYP2C9, CYP2D6 and CYP3A4. The CYP enzyme inhibition profile was important for the early identification of possible significant drug interactions.

### 4.11. Statistical Analysis

All the data are expressed as mean ± SD of three independent experiments. The IC_50_ values were calculated by a nonlinear dose/response regression model using GraphPad Prism software version 6 for Windows (San Diego, CA, USA, http://www.graphpad.com/, accessed on 10 February 2020). The relative changes in the expression of the gene were analysed by the 2^−ΔΔCt^ method [37]. The Student’s paired *t*-test was used to calculate the *p* value and the significance was considered if *p* < 0.05.

## 5. Conclusions

The ink concentrate of *B. leachii* exerts anti-proliferative and pro-apoptotic activities in the human liver cancer HepG2 cell line, suggesting *B. leachii* ink concentrate as a promising, safe, natural-based, neo-adjuvant drug for liver cancer treatment. Our computational predictions for the *B. leachii* ink concentrate-derived identified bioactive molecules suggest that these compounds have promising anti-cancer properties with acceptable drug-likeness profiles and minimal CYP enzyme inhibitions, which warrants further optimization and development to discover novel drug entities from marine-derived natural resources. Additional chemical isolation and in vivo studies are still required.

## Figures and Tables

**Table 1:** The bioactivity scores of identified metabolites from *B. leachii* ink concentrate using the PASS online webserver.

Anti-Neoplastic Activity	Probability of Being Active (Pa)	Probability of Being Inactive (Pi)
Hectochlorin	0.933	0.002
Malyngamide X	0.295	0.231
Malyngamide S	0.747	0.019
Bursatellin	not applicable	not applicable
Lyngbyatoxin A	0.169	0.075

**Table 2:** Molecular Target Predictions for bioactive molecules identified from *B. leachii* ink concentrate using Molinspiration and SwissTargetPrediction.

Name	Target Prediction (Molinspiration)		Target Prediction (SwissTargetPrediction)
Hectochlorin	GPCR ligand	−0.09	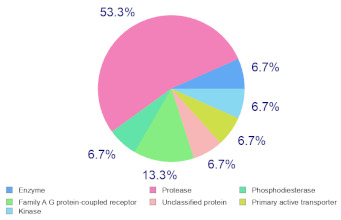
	Ion channel modulator	−0.46	
	Kinase inhibitor	−0.46	
	Nuclear receptor-ligand	−0.18	
	Protease inhibitor	0.1	
	Enzyme inhibitor	−0.08	
Malyngamide X	GPCR ligand	0.19	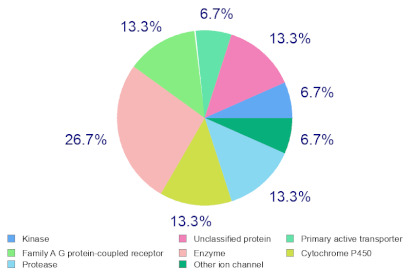
	Ion channel modulator	−0.44	
	Kinase inhibitor	−0.42	
	Nuclear receptor-ligand	−0.29	
	Protease inhibitor	0.46	
	Enzyme inhibitor	0.01	
Malyngamide S	GPCR ligand	0.17	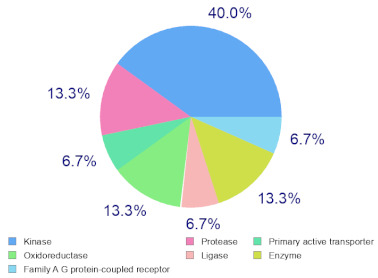
	Ion channel modulator	0.24	
	Kinase inhibitor	−0.28	
	Nuclear receptor ligand	0.23	
	Protease inhibitor	0.32	
	Enzyme inhibitor	0.32	
Bursatellin	GPCR ligand	0.04	NA
	Ion channel modulator	−0.22	
	Kinase inhibitor	−0.36	
	Nuclear receptor-ligand	−0.21	
	Protease inhibitor	0.01	
	Enzyme inhibitor	0.32	
Lyngbyatoxin A	GPCR ligand	0.49	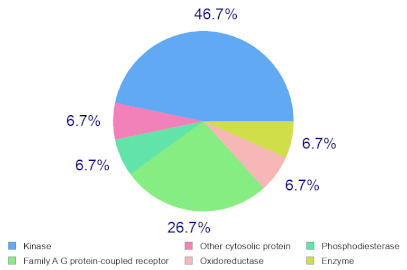
	Ion channel modulator	0.2	
	Kinase inhibitor	0.42	
	Nuclear receptor ligand	0.06	
	Protease inhibitor	0.36	
	Enzyme inhibitor	0.35	

**Table 3:** The predicted ADME properties for the bioactive molecules identified in *B. leachii* ink concentrate.

Compound Name	Molecular Weight	Log Po/w (WLOGP)	Log S (SILICOS- IT)	BBB Permeant	GI Absorption	Rule of Five (ROF)
Hectochlorin	665.60 g/mol	5.09	−6.86 Poorly soluble	No	Low	No; 2 violations: MW > 500, NorO > 10
Malyngamide X	607.82 g/mol	3.98	−5.07 Moderately soluble	No	High	Yes; 1 violation: MW > 500
Malyngamide S	484.07 g/mol	4.73	−6.25 Poorly soluble	No	High	Yes; 0 violations
Bursatellin	264.28 g/mol	−0.21	−2.39 Soluble	No	High	Yes; 0 violations
Lyngbyatoxin A	437.62 g/mol	4.09	−6.69 Poorly soluble	Yes	High	Yes; 0 violations

**Table 4:** The CYP enzyme inhibition profile of the bioactive molecules identified in *B. leachii* ink concentrate.

Compound Name	CYP1A2 Inhibitor	CYP2C19 Inhibitor	CYP2C9 Inhibitor	CYP2D6 Inhibitor	CYP3A4 Inhibitor
Hectochlorin	No	No	No	No	No
Malyngamide X	No	Yes	No	No	Yes
Malyngamide S	No	Yes	No	Yes	Yes
Bursatellin	No	No	No	No	No
Lyngbyatoxin A	No	Yes	Yes	No	Yes

## Data Availability

The data presented in this study are available on request from the corresponding author.

## Abbreviations

ADME	absorption distribution metabolism excretion
ATP	adenosine triphosphate
BBB	blood–brain barrier
cDNA	complementary deoxyribonucleic acid
CO_2_	carbon dioxide
DMEM	Dulbecco’s Modified Eagles Medium
FBS	foetal bovine serum
GAPDH	glyceraldehyde 3-phosphate dehydrogenase
GI	gastrointestinal
HCC	hepatocellular carcinoma
HIV	human immunodeficiency virus
IC_50_	half-maximal inhibitory concentration
Log P	lipid solubility
Log S	water solubility
mRNA	messenger ribonucleic acid
QTOF	quadrupole time of flight
ROF	rule-of-five
RT-qPCR	reverse transcription-quantitative polymerase chain reaction
STS	staurosporine
